# Heterotopic Ossification: A Comprehensive Review

**DOI:** 10.1002/jbm4.10172

**Published:** 2019-02-27

**Authors:** Carolyn Meyers, Jeffrey Lisiecki, Sarah Miller, Adam Levin, Laura Fayad, Catherine Ding, Takashi Sono, Edward McCarthy, Benjamin Levi, Aaron W James

**Affiliations:** ^1^ Department of Pathology Johns Hopkins University Baltimore MD USA; ^2^ Department of Surgery University of Michigan Ann Arbor MI USA; ^3^ Department of Orthopaedic Surgery Johns Hopkins University Baltimore MD USA; ^4^ Department of Radiology Johns Hopkins University Baltimore MD USA; ^5^ UCLA and Orthopaedic Hospital Department of Orthopaedic Surgery and the Orthopaedic Hospital Research Center Los Angeles CA USA

**Keywords:** HETEROTOPIC BONE, ECTOPIC BONE, MYOSITIS OSSIFICANS, FIBRODYSPLASIA OSSIFICANS PROGRESSIVA

## Abstract

Heterotopic ossification (HO) is a diverse pathologic process, defined as the formation of extraskeletal bone in muscle and soft tissues. HO can be conceptualized as a tissue repair process gone awry and is a common complication of trauma and surgery. This comprehensive review seeks to synthesize the clinical, pathoetiologic, and basic biologic features of HO, including nongenetic and genetic forms. First, the clinical features, radiographic appearance, histopathologic diagnosis, and current methods of treatment are discussed. Next, current concepts regarding the mechanistic bases for HO are discussed, including the putative cell types responsible for HO formation, the inflammatory milieu and other prerequisite “niche” factors for HO initiation and propagation, and currently available animal models for the study of HO of this common and potentially devastating condition. © 2019 The Authors. *JBMR Plus* published by Wiley Periodicals, Inc. on behalf of American Society for Bone and Mineral Research.

## Introduction

Heterotopic ossification (HO) is a diverse pathologic process, defined as the formation of extraskeletal bone in muscle and soft tissues. The word “heterotopic” is derived from the greek roots “hetero” and “topos,” meaning “other place.” HO can be conceptualized as aberrant tissue repair and is increasingly recognized as a common complication of trauma, surgery, and other local or systemic insults. Nongenetic forms of HO are most common, but rare genetic forms of HO also exist. The spectrum of HO is broad. Some HO lesions may be small and clinically irrelevant, while others may exact a high morbidity. HO is most commonly incited upon tissue injury, followed by an influx of inflammatory cells and subsequent downstream signaling sequelae among predominantly resident cells of mesenchymal origin. These downstream signaling events inappropriately activate an osteogenic or osteochondrogenic program. In practice, the designation of HO is applied to bone formation at any extra‐osseous site, including skeletal muscle, fascia, tendon, ligament, subcutis, skin, vascular wall, or virtually any site of connective tissue. Accompanying this anatomic/regional heterogeneity, the cellular contributions to HO formation and propagation are likewise diverse, made apparent by basic research studies utilizing lineage‐specific reporter animals. Even the basic mechanisms of bone formation are nonhomogeneous and may be either via intramembranous or endochondral pathways. In the present comprehensive review, we seek to synthesize the clinical, pathoetiologic, and basic biologic features of HO to both present a unified description of the current knowledge of HO and a reflection of its diversity in presentation, in etiopathogenesis, and in ongoing research efforts.

## Clinical Features of Heterotopic Ossification

### Epidemiology

The classic presentation of nongenetic HO is in young adults with a clear history of local trauma or surgery.^1^ Approximately half of patients are in their second and third decades of life; however, a broad age distribution is present from infancy to late adulthood.^2^, ^3^, ^4^, ^5^, ^6^ Men are slightly more commonly affected with a sex ratio of 3:2. A history of trauma as the initiating event is present in most cases (up to 75%),^2^, ^4^, ^6^ and unrecognized or “microtrauma” or repetitive mechanical stress is generally thought to be present in the remaining patients.

HO is well documented to occur at increased frequency with certain predisposing conditions, including orthopedic surgery, most commonly hip arthroplasty (occurs in up to ∼40% of cases^7^, ^8^, ^9^), bone fracture or dislocation (occurs in up to ∼30% of cases,^10^ with elbow trauma or dislocation being a common site of involvement^11^), high‐energy extremity trauma,^12^ traumatic brain and spinal cord injury and other neurologic disorders (occurs in up to ∼50% of spinal cord injuries^13^), and severe burns (occurs in up to ∼20% of third‐degree burns^14^). For severe traumatic amputations, this incidence rises to above 90%^15^). These predisposing factors are discussed in more detail below).

### Clinical presentation

Nongenetic HO can occur nearly anywhere in the body, but the most common areas include locations that are susceptible to trauma, such as the elbow, thigh, pelvis, and shoulder.^16^, ^17^ The head and neck is also a well‐described location for traumatic HO.^18^, ^19^ HO may occur in the skin, particularly in autoimmune disorders such as dermatomyositis^20^ and systemic sclerosis.^21^ The digits are also a well‐described site for HO, in which case the term “fibro‐osseous pseudotumor of the digits” is also used.^22^ A spectrum of other distinctive reactive bony lesions of the hands and feet are also well described (including florid reactive periostitis, subungual exostosis, and bizarre parosteal osteochondromatous proliferation). All have variably overlapping histologic features with HO (see McCarthy and Sundaram^23^ for a review); however, these lesions are almost always associated with the periosteum. As we will discuss, HO classically forms without connection to the periosteum and can later fuse to the periosteum as a secondary feature. Some anatomic sites are relatively infrequently involved by HO (for example, the viscera in both genetic and nongenetic HO,^24^, ^25^ or the diaphragm in genetic forms of HO^24^). Understanding why these tissue sites are relatively inhospitable to HO formation is an interesting and unanswered question in the field.

The clinical presentation depends on the temporal stage of nongenetic HO development. In the early/inflammatory phase, HO presents with localized pain, tenderness, and swelling. During this time, HO is often characterized by a rapid increase in size, which may arouse clinical suspicion of a soft tissue sarcoma.^5^, ^16^ In later stages and with gradual maturation of the bone tissue, the swelling becomes more localized, firm, and when adjacent to a joint may restrict motion. Lesions resembling HO have been reported within nerves^26^ or the abdominal mesentery and fascia,^27^ and their presentation is site specific. A unifying theme of all locations is the presence of connective tissue and thus of tissue sites that may contain stromal cells with osteogenic potential.

The rare genetic causes of HO have a different presentation and clinical severity than the far more common nongenetic cases, and include fibrodysplasia ossificans progressiva (FOP) and progressive osseous heteroplasia (POH). FOP is a rare, slowly progressive disorder caused by *ACVR1* mutations and initially presenting in childhood^28^, ^29^, ^30^ (OMIM:135100). Multiple congenital skeletal malformations are associated with FOP, including most frequently an abnormal first toe,^31^ dysmorphologies affecting the digits of the hand,^32^ and malformations of the cervical spine.^32^ FOP patients eventually develop progressive, painful flares and heterotopic lesions limiting mobility and function. Biopsies should not be performed on FOP patients because any surgical intervention leads to additional spread of heterotopic lesions. Most cases arise from a spontaneous mutation, but autosomal dominant transmission has also been described.^32^ FOP is characterized by progressive ossification of muscle, tendon, aponeuroses, and ligaments. Ossifications generally develop from cranial to caudal and axial to appendicular. Eventual peri‐articular and soft tissue ossification becomes so severe as to lead to difficulty with posture, gait, and respiration.^24^, ^33^, ^34^, ^35^ Median age at death is approximately 40 years.^34^, ^36^ The mechanisms of ACVR1/ALK2 mutations have been well documented, which includes the R206H mutation resulting in hyperactive bone morphogenetic protein (BMP) signaling and primarily endochondral ossification.^37^, ^38^, ^39^ Cells with the R206H mutation respond to Activin A with increased SMAD1/5/8 phosphorylation comparison with wild‐type cells.^40^ The bone formed is thought to occur through an endochondral process based on human data and animal models.

POH is a more recently characterized genetic form of progressive HO caused by heterozygous inactivating mutations in the *GNAS1* gene.^41^ POH is an autosomal dominant disorder and can be a spontaneous/new mutation in the affected person or paternal inheritance of the mutant allele (OMIM:166350).^41^, ^42^ Ossification in POH has a predilection for the skin and subcutis and appears to be primarily intramembranous, although sporadic cartilage may also be found. The molecular defect causing POH is the same as that causing pseudopseudohypoparathyroidism (PPHP) (OMIM: 612463),^43^ which has a constellation of physical findings referred to as Albright's hereditary osteodystrophy (AHO).^42^

### Radiography

**Figure 1:**
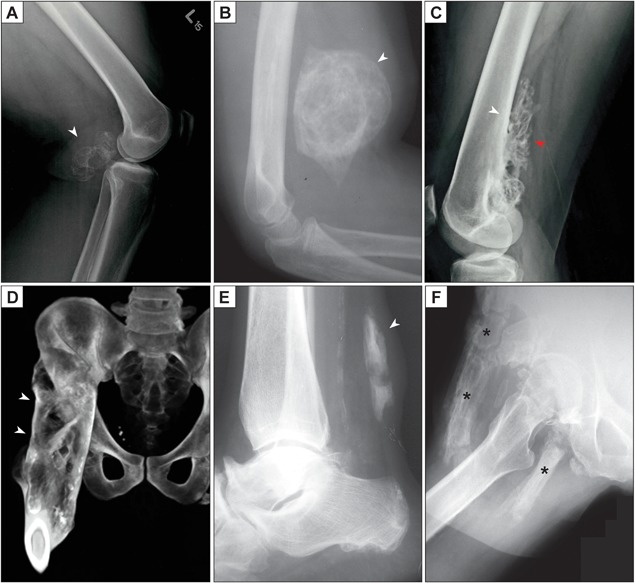
Radiographic appearance of heterotopic ossification (HO). (*A*) Lateral radiograph of the knee, demonstrating a posterior, well‐circumscribed soft tissue mass with peripheral radiodensity characteristic of HO (white arrowhead). A history of blunt trauma to the area was provided. (*B*) Lateral radiograph of the elbow, demonstrating an anterior, well‐circumscribed, and heavily ossified soft tissue mass (white arrowhead). Peripheral radiodensity is less apparent in this example. A history of antecedent local trauma was present. (*C*) Later, HO may form focal or even more diffuse connections to the underlying bone. In this unusual case, a well‐demarcated osseous lesion (red arrowhead) has a broad base of attachment (white arrowhead) to the posterior aspect of the distal femur. This lesion came to clinical attention due to local discomfort but without a history of trauma. (*D*) In severe cases, complete fusion (ankylosis) of the joint is observed, as is the case in the CT reconstruction with broad‐based connections of bone from the proximal femur to the ilium (white arrowheads). Clinical data not available. (*E*) Lateral radiograph of the ankle, demonstrating ossification of the Achilles tendon (white arrowhead). A history of local trauma was provided. (*F*) In this case of genetic heterotopic bone (fibrodysplasia ossificans progressiva), extensive ossification (asterisks) is present within the subcutis, muscle, and deep soft tissue around the proximal femur.

Radiographs are most often the first imaging study used to detect nongenetic HO and often have distinctive features that allow diagnosis. Unusual roentgenographic findings should prompt a second imaging modality. In the early phases of HO, no ossification can be found by radiographs.^44^ The radiographic appearance of HO is phasic and dynamic, which reflects the sequence of changes reflecting bony maturation. The classic appearance of mature intramuscular HO is that of a well‐developed and well‐demarcated radiodense mass, with a zonal ossification process (Fig. 1 *A*, *B*). Here, radiodensity is most apparent in the periphery of the lesion, imparting a calcified outline or shell to the mass, also termed “eggshell calcification.” Early lesions may have flocculent, irregular opacities without a clear zonal maturation pattern. HO most often involves the soft tissue only but may attach to the bone surface (also termed parosteal HO). HO attachment to the underlying bone is usually focal, but in longstanding lesions, a more broad‐based bony stalk to the underlying cortex may be found (Fig. 1 *C*). In parosteal HO, the periosteal reaction may obscure the classic clinicopathologic presentation. In advanced stages, the HO may be so massive as to cause complete ankylosis of the affected joint (Fig. 1 *D*). Several site‐specific or disease‐specific radiographic features of HO are also notable and deserve special mention. HO within tendons and ligaments has a distinctive appearance on radiographs that often follows the anatomic structure of the tissue (Fig. 1 *E*). The differentiation of tendinous calcification versus ossification by standard radiographs is not reliable, as a pseudotrabeculation pattern may be visible with calcification.^45^ HO in dermatomyositis often has a distinct roentgenographic appearance, with prominent stippled calcifications in clumped masses and sheetlike confluences.^46^ Distinct radiographic appearances in the rare genetic forms of HO have also been described.^47^ For example, radiographs of children with FOP generally show well‐circumscribed areas of deep HO that often corresponded to a distinct skeletal muscle (Fig. 1 *F* depicts more advanced ossification in FOP). In contrast, radiographs of children with POH showed a “cocoon‐like web” of HO entangling the connective tissues from the dermis down to the skeletal muscles.^47^

The appearance of HO by computed tomography (CT) is usually characteristic, and the zonal maturation of the lesion is well appreciated on CT.^23^ Again, the imaging characteristics of nongenetic HO in an intramuscular location are best characterized. Early in HO progression, a low‐density mass may be the only sign of HO by CT. In such cases and if the diagnosis of HO is suspected, short‐interval follow‐up CT imaging is helpful in making the final diagnosis as peripheral ossification develops. Cross‐sectional imaging with CT is also important in preoperative planning, by improving visualization of the lesion's relationship to important anatomic landmarks.^48^

By MRI, the appearance of HO is variable, depending on the stage of maturation. Typically, HO is a well‐defined mass with heterogeneous signal, characteristically associated with diffuse surrounding perilesional edema.^49^ In the early phases, there may be fluid‐fluid levels due to the presence of hemorrhage. Enhancement after contrast administration can occur centrally in HO due to vascularity within the lesion, making the differentiation from sarcoma challenging at times. However, the developing zonal ossification pattern is important in distinguishing HO from a sarcoma, as identified by correlative radiograph or CT. As HO matures, rim enhancement after contrast administration is the predominant feature of HO lesions, which helps to differentiate HO from a soft‐tissue sarcoma.^50^

Several site‐specific classification schemes exist to grade the severity of HO, which are particularly relevant for peri‐articular HO. The Brooker scale classifies hip‐associated HO into four classes of ascending severity (I–IV), which are related to the distance between HO and the hip joint.^7^ The Hastings and Graham classification scale for elbow joint–associated HO uses a three‐point functional scale (I–III) to define the degree of clinical and radiographic severity.^51^

Alternative imaging modalities may also be of benefit in the detection of HO. Positron emission tomography (PET) may be combined with CT, either using radiolabeled fluoride (F18) or radiolabeled glucose (FDG). F18 binds hydroxyapatite and detects areas of bone formation, and may be useful in the detection of nongenetic HO^52^ and recently in the early detection and monitoring of flare‐ups in FOP.^53^ FDG PET localizes to areas of increased metabolic activity and inflammation, and increased FDG avidity (although nonspecific) has been noted in cases of nongenetic HO.^54^ Single‐photon emission CT (SPECT) is a potential imaging modality for early detection of HO with potentially improved sensitivity.^54^, ^55^, ^56^ Although operator dependent, ultrasound can be used to detect HO especially in spinal cord injury patients.^57^, ^58^ Raman spectroscopy is a novel imaging technology that has the potential to define the extent of HO earlier than currently available radiographic studies by detecting mineralized collagen within tissues.^(59)^ Near infrared imaging and ultrasound imaging have also been described to identify HO before radiographic detection.^60^, ^61^

### Pathology

**Figure 2:**
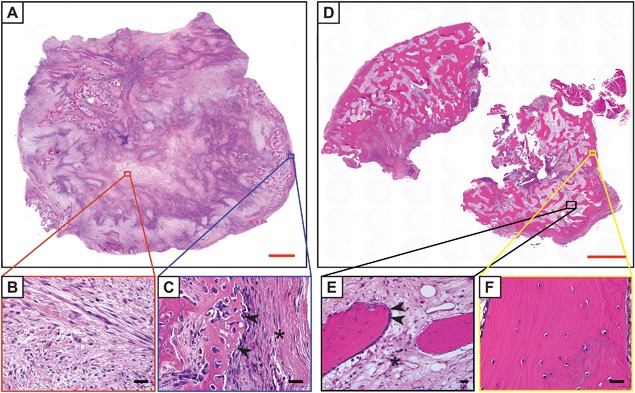
Histologic appearance of heterotopic ossification (HO) by H&E staining. (*A*) Whole‐mount, tile scan image of early HO, demonstrating a highly cellular lesion with multifocal areas of wispy osteoid formation. This enlarging intramuscular lesion was removed from the back of a 15‐year‐old patient, with no known history of local trauma. (*B*) Representative central area, which demonstrates a “granulation tissue‐like” spindle cell proliferation without bone. Magnification ×40. (*C*) Representative peripheral area, demonstrating woven bone (arrowheads) and compressed fibrous pseudocapsule on the lesions’ exterior (asterisk). Magnification ×40. (*D*) Mature HO, often received in fragments owing to the density of the bone tissue. Whole‐mount, tile scan image. Here, increased quantity of thickened, lamellar bone is observed. Longstanding intramuscular mass was excised from the forearm of a 31‐year‐old patient. No history of trauma or other predisposing factor to HO was obtained in this case. (*E*) Representative central area, with lamellar bone trabeculae with bone‐lining cells (arrowheads) with intervening fibrous tissue resembling fibrotic marrow (asterisk). Magnification ×20. (*F*) Representative peripheral area, with thickened lamellar bone bearing resemblance to native cortical bone. Magnification ×40. Red scale bars = 2 mm. Black scale bars = 25 µm.

**Figure 3:**
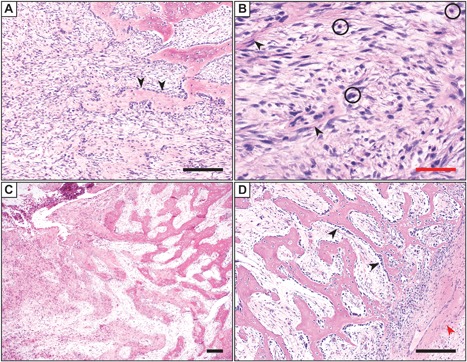
Histologic appearance of early/evolving heterotopic ossification (HO) by H&E staining. (*A*) The lesion is highly cellular and composed of spindle cells and scattered inflammatory cells. In the upper right, heterotopic bone formation is observed, with prominent osteoblast rimming (arrowheads). Note the intramembranous origin of bone in this case, which forms directly from stromal cell condensates. Magnification ×10. (*B*) Higher magnifications demonstrate the spindle cell proliferation in areas without bone formation. A cellular proliferation of spindled to ovoid cells are set in a variably edematous to fibrous stroma, with slender and elongated capillary‐type vessels (arrowheads) and a background of inflammatory cells (circles). No frank nuclear atypia or atypical mitotic figures are found. Magnification ×40. (*C*) A gradual continuum of woven bone (left) to more mature lamellar bone (right) is often observed in cases of nongenetic HO, which in an extraskeletal location is pathognomonic for HO. Magnification ×4. (*D*) Prominent bone lining osteoblasts are found (black arrowheads) in this example of HO. Also present in this image is the fibrous capsule around the periphery of the lesion (lower right), which often houses thick‐walled feeder vessels (red arrowhead). Magnification ×10. Images from parts *A*, *B*, and *D* are taken from the same case: a 6‐year‐old patient with an intramuscular pelvic mass and no additional clinical history. Images from *C* are from a 12‐year‐old patient with an intramuscular paraspinal mass. No additional clinical history available. Black scale bar = 100 µm. Red scale bar = 25 µm.

**Figure 4:**
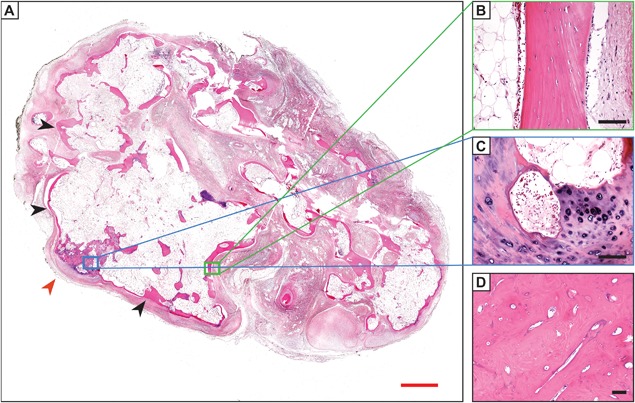
Histologic appearance of mature/late‐stage heterotopic ossification HO by H&E staining. (*A*) Appearance of mature HO, with thickened peripheral bone (black arrowheads), interior fatty marrow cavity (black asterisk), and foci of endochondral ossification (red arrowhead). (*B*) A neo‐cortex develops with elements resembling native cortical bone. Note an adipocyte‐rich interior on the left‐hand side of the image. (*C*) Focal metaplastic cartilage and endochondral ossification within this case are present. (*D*) In less common instances, sclerotic bone may develop, as seen in an osteoma. Images from *A–C* obtained from a 27‐year‐old patient with ossified soft tissue mass of the foot, with history of antecedent trauma; *D* obtained from a 66‐year‐old patient with an ossified soft tissue mass of the foot. Antecedent trauma as well as a history of previous resections was provided. All prior resections contained heterotopic bone. Red scale bars = 2 mm. Black scale bars = 50 µm.

Nongenetic HO is often designated by the tissue type it involves, such as myositis ossificans when involving skeletal muscle, or fasciitis ossificans when involving fascia. Myositis ossificans is the most common term used among pathologists, although this term is a misnomer when more broadly used to discuss HO (as HO is neither specific to muscle nor involves prominent inflammation after its early stages). Nevertheless, the term myositis ossificans is still commonly used. The histopathologic appearance of HO evolves over time (Fig. 2). The histopathologic features of nongenetic HO within an intramuscular location are most well described and are summarized below.^62^ A discussion of how the histopathology of HO differs based on other common tissues of origin, as well as differences between genetic and nongenetic HO, is described in subsequent paragraphs. Early lesions are often hypercellular and with little bone matrix, and can prompt concern for a soft‐tissue sarcoma. Later lesions have prominent bone formation with a characteristic zonal architecture that is best appreciated a low magnification (Fig. 2). This zonal architecture with a predominant peripheral ossification is a hallmark of HO. Early lesions demonstrate a hypercellular proliferation of spindle cells often with little bone matrix (Fig. 3 *A*, *B*). Spindled areas often have features of “nodular fasciitis” or “granulation tissue,” including high numbers of normal mitotic figures, scattered multinucleated giant cells, scattered inflammatory cells, and extravasated red blood cells. As ossification ensues, woven bone with prominent osteoblastic cell lining is characteristic (arrowheads, Fig. 3 *A*). A gradual continuum of woven bone to more mature lamellar bone is often found, one helpful feature to distinguish HO from extraskeletal osteosarcoma (Fig. 3 *C*). HO is well circumscribed, with a surrounding fibrous pseudocapsule often with thick‐walled blood vessels (Fig. 3D).^63^ More mature lesions resemble native bone elements, including thickened trabeculae of lamellar bone with central fatty marrow and vascular spaces resembling bone marrow sinusoids (Fig. 4 *A*). The peripheral aspects of mature HO may demonstrate compact bone tissue, which resembles native cortical bone (Fig. 4 *B*), sometimes including histologic features reminiscent of Haversian systems and Volkmann's canals. Among nongenetic intramuscular HO, metaplastic cartilage and endochondral ossification may be found but are usually focal and may not be present (Fig. 4 *C*). As we will discuss in later paragraphs, HO lesions may demonstrate more frank cartilage in a site‐specific and disease‐specific manner. Other cases may demonstrate areas of dense, sclerotic bone as found in an osteoma (Fig. 4 *D*).

For the practicing pathologist, the most important diagnostic distinction is between HO and extraskeletal osteosarcoma (OS). Helpful histologic findings of HO include presence of bone “maturation” and spatial zonation with more peripherally mature bony elements.^63^ The zonation phenomenon is most conspicuous in lesions involving skeletal muscle (myositis ossificans) and are less frequently found in nongenetic HO involving fascia or the subcutis.^64^ Importantly, the microscopic features of immature bone trabeculae with osteoblastic rimming and a progression to more mature bone are not found in osteosarcoma.^65^ As well, the absence of frankly sarcomatous features is an important diagnostic distinction. HO does not have obvious nuclear atypia or atypical mitotic figures.^66^ Occasionally HO with connection to the bone surface may pose a diagnostic challenge to osteosarcomas that arise on the bone surface (periosteal or parosteal OS^65^). Any challenging biopsy that presents a diagnostic dilemma should be reviewed in the context of clinical and radiographic findings, with a low threshold for consultation.

Striking histologic similarity is present between fracture healing and HO, which deserves special mention. Like early fractures, early HO demonstrates a fibroproliferative stroma with mitotic activity. Like fractures, bone formation is via a variable combination of intramembranous and endochondral bone. As HO matures, woven bone gives way to lamellar bone, which over time is remodeled to develop a cortical appearance. Bone marrow components are recruited into intervening bony elements of HO and gives rise to the normal elements of bone marrow, including a multilineage marrow, adipocytes, and osteoclasts. As bone maturation occurs, so too does the vasculature within HO develop and mature. Capillary‐like vessels in early HO lesions, also found in granulation tissue‐like areas of fractures, give rise to bone marrow sinusoid type vessels in later HO.^63^

Ossification within tendons and ligaments have an overlapping but distinct histopathologic appearance from HO as previously discussed.^67^, ^68^ Ossification within tendons may occur within the tendinous body or the connection to the bone (enthesis). The abnormal ossification of tendons, also termed tenosynovial chondro‐osseous metaplasia, can either follow acute trauma or chronic inflammatory insults (eg, spondyloarthritic ossification and “tendinitis”).^67^, ^69^ HO within tendons is less frequently examined on a histopathologic level, as the morbidity of tendon resection is high. Nevertheless, it seems that endochondral ossification may be a more prominent feature in tendinous HO than in classic intramuscular HO,^70^, ^71^, ^72^, ^73^, ^74^ although both intramembranous and endochondral tendon HO have been reported.^72^ This is not surprising given the well‐defined expression of the chondrogenic transcription factor SOX9 within the entheses.^75^ Foley and colleagues recently reviewed the histologic features of peri‐articular HO, and it appears that an endochondral pathway is also a common and distinguishing feature in this location.^76^ These observations are supported by animal studies that reproduce a robust endochondral HO with tendinous injury.^77^, ^78^ Dystrophic calcification is more commonly observed within HO of the tendon or enthesis rather than intramuscularly (sometimes termed calcifying tendinopathy), and calcification may be admixed with ossification.^70^, ^71^, ^79^ The “mature” bony lesions of HO look essentially identical irrespective of their tissue location of origin^68^ and are essentially indistinguishable from normal bony elements.

The basic pathways of bone formation in HO are somewhat controversial and deserve special emphasis. Nongenetic HO can form through both endochondral and intramembranous ossification processes, and it is the authors’ opinion that a spectrum exists within a given lesion from “endochondral‐predominant” HO in some cases to “intramembranous‐predominant” HO in others. Classic intramuscular HO (termed myositis ossificans), which is biopsied early in its evolution, almost always shows intramembranous rather than endochondral bone, and indeed frank cartilage is rare.^1^, ^23^, ^62^ In contrast and as discussed, Foley and colleagues recently found cartilage and endochondral ossification in all sampled cases of peri‐articular HO.^80^ Other examples of tissue depot–specific variability exists as well, where, for example, dermal HO may be more often intramembranous in quality,^81^ whereas tendon‐associated HO in animal models^77^ or juxta‐articular HO in human patients may be more often endochondral.^80^ This variability is compounded by the phasic nature of HO, in which the cartilaginous template of endochondral HO may be missed on biopsy or resection.

As previously alluded to, the rare genetic forms of HO do have distinguishing histologic characteristics. FOP is a predominant endochondral process,^82^ and in the authors’ clinical experience, the amount of ossifying cartilage in FOP greatly exceeds that of typical nongenetic HO. In contrast, POH is a predominant intramembranous process with sporadic foci of frank cartilage observed in some cases.^83^ Whether the intramembranous‐predominant process in POH is owing to the common dermal location or rather the underlying molecular etiology remains unclear. Dystrophic calcification (DC) leading to ossification is a third potential mechanisms for HO. Human evidence for this transition or mixture of DC and HO has been described in tendinous HO^70^, ^71^, ^79^ and in dermatomyositis (where abundant DC is often mixed with HO).^20^

### Clinical risk factors: mechanism of injury

#### Hip arthroplasty

**Figure 5:**
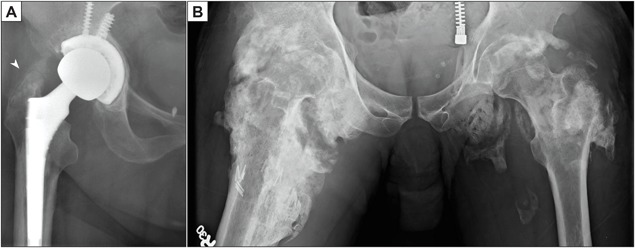
Examples of HO post arthroplasty or spinal cord injury (neurogenic HO). (*A*) Flocculent radiodensity representing developing HO superior to the greater trochanter (arrowhead) in a post‐arthroplasty patient. (*B*) Massive and bilateral, peri‐articular HO in an immobilized patient with spinal cord injury.

After total hip arthroplasty (THA), between 2% and 7% of patients develop extensive periarticular HO^84^ (Fig. 5 *A*). In fact, when all severities of ossification are included, HO may occur at up to 40% post arthroplasty.^7^, ^8^, ^9^ This is even more common in secondary hip replacements. Patients with ankylosing spondylitis, Paget's disease, and hypertrophic osteoarthritis are at risk of developing HO post arthroplasty.^85^ Certain surgical factors also predispose to HO post arthroplasty, including extended ischemia time, use of cemented implants, and type of approach.^86^ In general, minimally invasive surgery (MIS) approaches (such as MIS anterolateral [MIS‐AL] and minimally invasive direct anterior approach [AMIS]) are thought to carry a lower risk of HO than the standard modified anterolateral (STD‐Watson‐Jones) approach.^87^ However, only a few studies have been done relating surgical approach to HO, and whether MIS approaches are more effective than transgluteal STD‐Bauer approach is still unknown.^87^, ^88^ Surgeons who perform a posterior approach may resect the gluteus minimus to decrease risk of HO.^89^

#### Fractures

The formation of heterotopic ossification after orthopedic trauma has been studied most extensively in the setting of acetabular fractures and elbow fractures. Heterotopic ossification occurs in approximately 40% of patients after operative fixation of an acetabular fracture.^90^ The surgical approach can impact the risk for heterotopic ossification, possibly related to the amount of operative soft tissue damage.^91^ More than 20% of those who develop HO in this setting have clinically relevant range of motion deficits. Additional risk factors include concomitant neurologic injury, delayed internal fixation, and use of bone graft and/or bone‐graft substitute. Animal models of polytrauma‐induced HO have been developed, which mimic blast injury and/or major orthopedic intervention.^92^

#### Spinal cord and traumatic brain injuries

HO is well known to develop around joints after central neurologic injury, including traumatic brain injury and spinal cord injury (Fig. 5 *B*). The incidence of neurogenic HO has been reported to range from 10% to 53%.^13^ Patients with low cervical or high thoracic lesions are the most likely to develop this complication.^93^ Patients with severe spasticity, impaired cognition, tracheostomy, pneumonia, and/or urinary tract infections are at a higher risk.^93^ Patients with neurogenic HO develop lesions around larger joints.^23^ HO after spinal cord injury generally forms caudal to the level of injury, whereas HO after closed TBI may occur around any large joint, including the hip, knee, elbow, and shoulder.^94^ Involvement of small joints by neurogenic HO is rare.^23^ Development of neurogenic HO generally occurs within a few months after CNS injury and progresses over a period of years.

Although the relationship between the nervous system and the formation of bone remains incompletely understood, it is known that peripheral neurotransmitters affect osteoblast formation.^93^, ^95^ Animal studies that denervate bone have resulted in significant alterations in bone metabolism and healing. For example, sciatic nerve dissection reduces bone growth and impairs fracture healing.^96^, ^97^ Similarly, mice treated with the vanilloid capsaicin to chemically denervate sensory nerves exhibited lower trabecular and cortical bone volumes.^98^, ^99^ It is not yet clear how central nervous system insults initiate a sequence of events that lead to juxta‐articular HO. Other contributing factors may include prolonged immobilization, vascular stasis, edema, as well as the passive manipulation of joints in immobilized patients. Nevertheless, animal models of spinal cord injury–induced HO have been devised, which incorporate a combination of spinal cord transection with intramuscular insult.^100^ Interestingly, the mechanism of ossification in CNS injury models of HO has not been described, and thus whether endochondral, intramembranous, or DC‐rich lesions are present is as yet unknown.

#### Thermal injury

HO is a well‐recognized complication of burn injury, with increasing risk of HO with increasing percentage of body surface area affected.^101^ Burns involving >20% of the body substantially increase the likelihood of HO formation.^101^ Additional risk factors include male sex and full‐thickness injury at or near a joint.^101^ Thermal injury–induced HO is similar to neurogenic HO in that it occurs with striking predilection for the juxta‐articular regions. HO occurs with the highest frequency at the elbow, followed by the shoulder, and subsequently the hip. Owing to its location around joints, those patients with burn injury–induced HO often have a restriction in the range of motion as an early manifestation of the ossification. Among burn patients, it is particularly important to distinguish juxta‐articular HO from scar contracture—both of which limit joint movement. Small animal models to re‐create burn injury–induced HO have been developed (combined burn + tenotomy model).^78^

### Management

The potential management options for HO are best divided into two categories: 1) prophylactic strategies to prevent or mitigate the extent of HO, and 2) treatment strategies to improve symptoms and function once the condition has occurred. Most studies pertain to non‐genetic HO, while specific treatments for genetic forms of HO are also discussed. The distinction is often determined by the presence or absence of HO at the time of intervention, though there may be a period of overlap in the early stages of ossification where the severity may be decreased. In a survey of orthopedic and trauma centers in Germany, two‐thirds of respondents reported routine use of prophylaxis against HO for high‐risk injury patterns.^102^

#### Prophylactic strategies

##### Radiation therapy

Low‐dose radiation has been studied both as a prophylactic modality in HO occurrence (as primary prevention in high‐risk patients) or as a prophylactic modality in HO recurrence (as secondary prevention together with surgical excision). The prophylactic use of radiation therapy is best studied in the context of hip arthroplasty. Of the randomized controlled trials that have been performed, definitions of high‐risk patients vary but may include those patients with hypertrophic osteoarthritis, ankylosing spondylitis, diffuse idiopathic skeletal hyperostosis, or prior HO.^103^ Thus, prophylaxis against HO occurrence (in those at high risk) and HO recurrence (preexisting HO) are often studied together. Prophylactic doses typically range from 400 to 800 cGy, and are given either 24 hours preoperatively or up to 72 hours postoperatively.^103^ For patients undergoing total hip arthroplasty, a single‐center randomized trial suggests that 700 cGy given postoperatively was significantly more effective at preventing HO (25%) than 400 cGy (42%)^104^ given postoperatively. Higher doses, however, have not proven to be of increased benefit for prevention.^105^ Furthermore, there does not appear to be a significant difference between preoperative and postoperative radiation dosing regarding efficacy or complications, with the exception that those treated more than 8 hours preoperatively or more than 72 hours postoperatively may demonstrate a greater rate of radiographic ossification after hip surgery.^105^, ^106^

Although the majority of controlled studies have focused upon patients undergoing surgery around the hip, the results regarding prophylactic radiation have been extended to other indications as well.^107^ Among spinal cord injury patients with evidence of early neurogenic HO, a single radiation fraction of 700 cGy limited the progression of ossification around the hips.^108^ After elbow trauma, however, the results are somewhat less favorable, with limited evidence to support its routine use.^109^ A randomized controlled trial was stopped early because of concerns over increased rates of nonunion in those receiving 700 cGy of radiation after surgical management of intra‐articular elbow fractures.^110^

Among the concerns with the use of prophylactic radiation are joint stiffness and potential oncogenesis. The relatively low risk of joint stiffness after low‐dose radiation can be weighed against the decreased range of joint motion and potential ankylosis that can develop with HO. Regarding oncogenesis, a case‐control analysis has failed to demonstrate a significantly increased rate of malignancy in patients treated at this low dose of radiation.^111^ It bears noting, though, that the number of patients needed for adequate power is rarely met, and the theoretical risk of malignancy remains.^112^

##### NSAIDs

Nonsteroidal anti‐inflammatory drugs (NSAIDs) remain the most commonly utilized prophylaxis for HO.^102^ Numerous NSAIDs have demonstrated efficacy, though postoperative indomethacin has been the historical gold standard, traditionally dosed at 25 mg three times daily for up to 6 weeks after surgery.^113^

In comparison to single‐dose radiation, indomethacin has shown equal efficacy in preventing HO after total hip arthroplasty and acetabular fracture surgery in high‐risk individuals.^85^, ^114^ However, the optimal NSAID duration and dosing regimen has not been definitively proven.^94^ Other studies have utilized ibuprofen, diclofenac, and ketorolac, among others. More recently, consideration has been given to the use of COX‐2 selective inhibitors, due to concerns over gastrointestinal effects with nonselective NSAIDs.^115^ In a case‐controlled study in total hip arthroplasty, celecoxib demonstrated significant reduction in the incidence of HO compared with untreated controls.^115^ When comparing COX‐2 selective inhibitors with nonselective NSAIDs, there does not appear to be a significant difference regarding their efficacy, nor their dose‐limiting toxicities over the short time period of prophylactic use.^116^

Numerous studies have suggested that NSAIDs may prove beneficial for indications unrelated to hip arthroplasty and acetabular fracture surgery, including hip arthroscopy, elbow trauma, and spinal cord injury.^117^, ^118^, ^119^ Despite its relatively low rate of complications, NSAID use has been associated with an increased risk for nonunion of acetabular fractures.^90^ Overall, the utility of NSAIDs and radiation in preventing nongenetic HO appear similar, though the cost associated with NSAID therapy is typically markedly less.^120^

##### Other prophylactic modalities

Although not commonly used in nongenetic HO, corticosteroids are used as a prophylactic modality in FOP patients. A brief course of high‐dose corticosteroids is often used in FOP patients within the first 24 hours of flare‐ups to reduce inflammation and tissue edema observed in early stages of the disease.^24^ However, the use of corticosteroids in FOP patients is generally limited to treating flare‐ups in the major joints, the jaw, and submandibular area. Corticosteroids are not used as a chronic treatment of FOP, so as to limit side effects associated with long‐term use.^121^

Despite early enthusiasm for the potential role of bisphosphonates in the prevention of neurogenic HO, further analysis has failed to demonstrate a clear benefit for the use of these medications in preventing HO.^122^, ^123^, ^124^ In fact, there is some indication that antiresorptive therapy may increase the risk of developing HO, or may simply delay rather than prevent the bone formation. Future therapeutic modalities such as retinoic acid receptor (RARγ) agonists^125^ (discussed in more detail in Signaling Pathways in HO below), as well as free‐radical scavengers^126^ are currently under investigation, though their clinical utility remains to be elucidated.

#### Treatment strategies

##### Physical therapy

It is unclear whether physical therapy plays a significant role in the development or mitigation of HO, and conflicting thoughts are reflected in the literature among burn and spinal cord injury patients about early range‐of‐motion exercises.^94^ For patients with contusions to the thigh, a quadriceps stretching regimen has been proposed as a method for improving the time to return to full activity and potentially preventing HO.^127^ In the authors’ clinical experience, burn surgeons will often comment on the increasing incidence of HO in those patients who receive overly aggressive passive range‐of‐motion exercises on the elbow in an attempt to prevent skin contracture. Direct comparison trials are lacking, and the applicability of stretching or range‐of‐motion exercises for other risk factors for ossification are questionable. In patients who have developed maturing HO, clinical management differs based on clinician preferences. For example, many recommend against passive exercises, which could exacerbate inflammation and possibly HO, while others recommend a physical therapy regimen to improve range of motion and limit contractures.

##### Surgery

The natural history of HO is to fully ossify into mature bone over time. Many patients with HO report pain, painful motion, restricted motion, or prominent bone that can lead to pressure sores or impaired hygiene. Although the focal discomfort may improve after the inflammatory stage abates, patients with persistent symptoms have few management options other than operative intervention.^102^ Patients without significant symptoms, however, can be managed nonoperatively.

Surgical resection for nongenetic HO is ideally performed after the osseous maturation is complete, which is typically by 6 months after the initiation of HO. Excision before 6 months may be associated with an increased risk of recurrence of HO.^128^ No additional benefit appears to exist with further delay in operative management.^128^, ^129^, ^130^ Although some have advocated that excision should be performed early to prevent irreversible loss of motion, a comparison between 18 patients with elbow ankylosis and 27 patients with partial restriction of motion demonstrated comparable return of motion.^131^ Especially for HO at specific anatomic sites (such as tendinous HO or intraperitoneal HO), the benefits of removal must be weighed against the morbidity of the surgical procedure itself. As previously alluded to, surgical management in FOP is generally not recommended because this may lead to additional spread of heterotopic lesions.

Even though complete excision is not always practical or possible, incomplete resection of the HO is associated with recurrence.^128^ Interposition of soft tissue is not of clear benefit.^102^ It is important to note that, unlike neoplastic processes, HO does not always respect natural anatomic barriers and may encase major neurovascular structures. This is perhaps responsible for the high reported rates of neurovascular injury after operative intervention in these patients.

## Basic Biologic Features of Heterotopic Ossification

### Animal models of HO

Many models have been devised to study heterotopic ossification. They can be roughly divided into those that mimic a FOP‐like phenotype via similar signaling cascades, those that induce HO by causing trauma, and those that mimic neurogenic HO formation via spinal cord injury. Many traumatic models use a combination of insults to generate significant HO.

#### Genetic models

Genetic models of HO formation typically replicate conditions similar to FOP. There are two major FOP‐like animal models of HO with mutations in the Acvr1 (Alk2) receptor: the Alk2 R206H^132^, ^133^, ^134^ and Alk2 Q207D^125^, ^135^ models. The Alk2 R206H mutation is most commonly used and can reproduce HO with a clinical presentation analogous to FOP.^132^ With global expression of the mutant allele, HO spontaneously occurs in the sternum, caudal vertebrae, hip joint, and hindlimb. As in human FOP, lesions progressively ossify and eventually fuse to native skeletal elements.^132^ Depending on the particular driver, both injury‐dependent and non‐injury‐dependent models exist for development of FOP‐like lesions.^132^, ^133^ These serve as preclinical tools and have been used for the ongoing development of treatments for FOP. In addition to global Cre models of Acvr1R206H, tissue‐specific drivers of Cre have been studied in FOP models and may be able to help determine cell types responsible for HO.^133^ These studies help support the putative cell types responsible for FOP‐like HO formation (discussed in more depth in Cell Precursers of HO below).

#### Traumatic models

Trauma‐induced animal models of HO have been developed. Early attempts applied blunt‐force trauma to muscle but had low success rates. Models that combine insults have had more consistent development of HO, such as blunt‐force trauma and a period of forced range of motion.^136^ A model of hip surgery has been developed in New Zealand white rabbits where an incision is made over the greater trochanter and the medullary canal of the femur is reamed out, leaving the bone reamings in the wound. Intentionally causing muscle injury by performing greater dissection, wider exposure, and clamping of the gluteus muscles to induce ischemia increases HO formation relative to minimal dissection and no clamping.^137^, ^138^ Another model of traumatic HO formation also demonstrates the benefit of multiple insults in causing consistent heterotopic bone formation. A mouse model demonstrates that 30% total body surface area (TBSA) burn in conjunction with Achilles tenotomy significantly increases heterotopic bone formation compared with tenotomy or burn alone.^59^

#### BMP‐induced models

Local injection or surgical implantation of BMPs and/or mesenchymal progenitor cells expressing BMPs can be used to induce HO formation.^139^ A variety of vehicles can be used to carry the BMPs, such as an injectable Matrigel or an implantable collagen sponge. There are a variety of options for where to implant the vehicle. Subcutaneous application is appealing for its technical ease, the lack of confounding osteogenic components in this space, and the space for implantation in lax rodent skin.^136^, ^140^, ^141^ Subcutaneous injection of BMPs in Matrigel is commonly employed.^141^ Impregnated Matrigel solidifies in vivo to form a localized source of BMP ligand.^142^ Intramuscular injection^143^ or implantation^144^ is also a good option for studying HO formation, although one must remove the confounding factors of osteogenesis in host muscle stromal cells and the upregulation of inflammatory and pro‐osteogenic signaling in response to the muscle injury itself.^145^ Currently, BMP‐induced models rely widely on recombinant human BMP2^142^ but have also studied other BMP family members including BMP4^146^ and 9.^145^ Kidney (infrarenal) capsule implants can be used to study HO formation, although this is less frequently done because of technical difficulty.^140^

#### Neurogenic and spinal cord injury models

A subset of the acquired or traumatic HO models, models of neurogenic HO have been developed. One such model uses a laminectomy and sharp transection of the spinal cord at the T7‐8 level with concomitant muscle inflammation provoked by injecting cardiotoxin into the hamstring muscle of the mice.^100^ This model again demonstrates the commonality of multimodal injury for development of reliable HO in an experimental setting.

**Table 1:** Summary of Local Cell Types That Have Been Observed to Directly Contribute to Murine HO Using Transgenic Reporter Mice

Cre driver	Cell source
Prx1‐Cre	Mesenchyme^150^
Nfatc1‐Cre	Mesenchyme^213^
Dermo1‐Cre	Mesenchyme^211^
Scx‐Cre; Scx‐CreERT2	Tendon/periosteum/fascia^149^, ^150^
Mx1‐Cre	Skeletal muscle interstitium/bone marrow^149^
Gli1‐CreER	Interstitial/perivascular cells^212^
Glast‐CreERT	Pericyte/adipocyte/connective tissue interstitium^153^
Wnt1‐CreERT	Endoneurium^154^
Tie2‐Cre/VE‐Cadherin‐Cre	Endothelium/muscle satellite cell^142^, ^155^

**Table 2:** Summary of Cell Types That Have Been Observed to Not Directly Differentiate Into Ectopic Bone and Cartilage in Murine HO Using Transgenic Reporter Mice

Cre driver	Cell source
Cadh5‐CreER^T2^	Mature endothelium^149^
CD19‐Cre	B cells^161^
LCK‐Cre	T cells^161^
LysM‐Cre	Monocyte/macrophage^161^
Nestin‐Cre	Somite‐derived cells^161^
Myf5‐Cre	Skeletal muscle^149^, ^161^
MyoD‐Cre	Skeletal muscle^142^
SM22a‐Cre	Vascular smooth muscle/pericyte^149^
SMMHC‐Cre	Vascular smooth muscle^142^, ^149^
NG2‐Cre	Arteriolar pericyte^214^
FoxD1‐Cre	Pericyte/connective tissue interstitium^153^
Vav1‐Cre	Hematopoietic cells and endothelium^149^

### Cell precursors of HO

Much has been learned from transgenic reporter animals to identify the cell types putatively responsible for bone and cartilage formation within HO. The data using transgenic reporter mice will be the focus of this review (Tables 1 and 2), although HO progenitor cell correlates have also been defined by flow cytometry in mouse models.^147^ Several caveats exist in the interpretation of lineage tracing studies, including:

1. Animal models of HO have some substantive differences in form of trauma, location of HO, and presence or absence of specific genetic mutation, and so not all models of HO would be expected to have the same cellular contributors.
2. Off‐target Cre expression may occur when Cre is expressed in cells or tissues not directly dictated by the promoter used to drive Cre expression. Individual reporters are often more broadly distributed than first reported.
3. Presence of the Cre transgene alone may result in cellular toxicity and unintended phenotypes that may secondarily impact the study of HO.
4. Some Cre strains demonstrate mosaic patterns of Cre expression that can differ widely between littermates. For example, this has been documented in Vav1‐Cre line,^148^ which has been studied in HO.^149^
5. Additional potential drawbacks exist in the case of ligand‐dependent (eg, Cre‐ERT system and tet‐ON/tet‐OFF systems) reporter systems. For example, background Cre activation in the absence of tamoxifen (leakiness) may occur with variable frequency depending on the construct. As well, the domain of reporter activity can be influenced by the ligand administration schedule, including the dose, route, and timing, as well as the chase period.
6. Finally, injury models of HO may induce reporter activity in an aberrant fashion in cells that natively do not normally express the factor.

Nevertheless, the aggregate data suggest that the predominant source of HO is generally from local stromal/fibroblastic cells of mesenchymal origin within the connective tissue of skeletal muscle, fascia, and/or subcutis. This includes use of Prx1‐Cre,^150^, ^151^ Scx‐Cre and Scx‐CreERT2,^(149,150)^ Mx1‐Cre,^149^ NFATc1‐Cre,^152^ and Glast‐CreERT^153^ strains that all have overlapping domains of distribution within reporter animals. Other putative cell types that may contribute to HO genesis include endoneurial cells highlighted by Wnt1‐CreERT,^154^ pericytes and other perivascular cells also highlighted by Glast‐CreERT reporter animals,^153^ and endothelium, which has undergone endothelial‐to‐mesenchymal transition as highlighted by the Tie2‐Cre mouse strain (so‐called EndMT).^142^, ^155^ In the case of potential direct endothelial contribution to HO, a mesenchymal population of Tie2+Pdgfra+Sca‐1+ cells resident in skeletal muscle may also represent the Tie2+ direct contribution to HO (and thus a non‐endothelial Tie2+ cell contributor may predominate).^156^ Several studies suggest that circulating mesenchymal cell types also contribute to HO formation,^157^, ^158^, ^159^, ^160^ which are likely nonhematopoietic in origin.^160^ Conversely, studies in reporter animals have suggested several cell types that likely do not represent direct cellular precursors of ectopic bone and cartilage in HO, including degenerating skeletal muscle fibers, vascular smooth muscle, and chronic inflammatory cells^142^, ^149^, ^161^ (Table 2). Nevertheless, and given the diversity of murine HO models, it is important to realize that which cell types directly contribute to HO bone and cartilage and which cell types represent requisite “niche” factors for HO genesis are both still not clearly defined.

### Inflammation and HO

In a simplistic conceptualization, inflammation is a key “niche factor” for the development of HO and a commonality across many of the conditions that predispose to HO formation. HO is known to occur in autoimmune diseases such as limited cutaneous systemic sclerosis,^21^ dermatomyositis,^20^ and inflammatory arthritis.^162^ Autoimmune diseases affecting the nervous system have also been reported as predisposing factors to HO, including anti‐NMDA receptor encephalitis^163^ and Guillain‐Barre syndrome.^164^ The role of trauma‐induced inflammation as inciting bone formation is logical, as inflammation characterizes the early stages of fracture repair.^165^ Use of NSAIDs for traumatic HO prophylaxis, and steroids for FOP treatment, rests on the theory that reducing postoperative inflammation will likewise reduce HO formation.^166^ Animal studies have begun to elucidate the complex and multifaceted role of the immune system in HO genesis and propagation.

#### Innate immunity and HO

Macrophages play an important role in endochondral ossification and fracture repair. Existing research likewise implicates the involvement of macrophages in several mouse models of HO^167^ and in human HO. Formation of HO in a BMP4 overexpression model has been shown to be dependent on macrophages, as macrophage depletion diminishes HO formation.^161^ Likewise, in a mouse model of neurogenic HO using spinal cord injury combined with muscle injury, investigators identified macrophages in HO tissue and determined that depletion of macrophages significantly reduced HO formation.^100^ This finding was reinforced in the Acvr1 R206H knock‐in mouse model of FOP.^168^ Macrophage infiltration was observed to occur at early stages of HO lesion formation, and macrophage depletion with Clodronate treatment reduced but did not completely prevent HO formation in this study.^169^ However, the route of administration for Clodronate (intraperitoneal versus intravenous) has different effects on monocytes, and it remains to be seen if more effective mechanisms to target the macrophage in HO can be developed. Accumulation of macrophages have likewise been observed in FOP patient samples.^169^ More lineage‐specific depletion methods and characterization of the monocyte subpopulations that traffic to the injury site are needed. As well, the cargo delivered or paracrine activity of macrophages in an HO setting have begun to be examined. For example, in a mouse model of neurogenic HO caused by spinal cord injury, Oncostatin M of macrophage origin was observed to promote osteoblastic differentiation of precursor cells.^170^ Thus, though macrophage infiltration of the soft tissue and presumed paracrine stimulation of HO formation is a consistent feature across genetic, traumatic, and neurogenic models of HO, further studies are needed to validate these findings.

Mast cell involvement in the pathogenesis of HO may also be important. Increased mast cells have been documented in cases of nongenetic HO, with mast cells appearing near sites of ectopic bone formation upon biopsies of HO at various sites.^82^ Increased mast cell density may be an even more prominent finding in FOP, with up to 150‐fold greater mast cell density at the periphery of FOP lesions compared with other inflammatory myopathies.^171^ Inhibition of mast cell degranulation can be accomplished by administration of Cromolyn, an FDA‐approved drug for asthma. Cromolyn treatment significantly reduced ectopic bone formation in a BMP2‐indcued mouse model of HO,^82^ as well as the Acvr1 Q207D transgenic mouse model of FOP.^172^ Additionally, the c‐kit tyrosine kinase inhibitor Imatinib induces mast cell apoptosis, and Imatinib treatment was observed to decrease HO in an Achilles tenotomy model of traumatic HO in mice.^82^ Interestingly, there is some evidence that mast cells and macrophages may in some circumstances synergize to induce HO formation.^169^ Methods to deplete both macrophages and mast cells reduced HO formation in the Acvr1 R206H knock‐in mouse model greater than depletion of either cell population alone.^169^

#### Adaptive immunity and HO

Like the innate immune system, the adaptive immune system seems to play a role in HO genesis and/or propagation. The precise mechanisms are not well understood. Lymphocytic inflammation has been described as a common histologic feature of HO,^161^ particularly perivascular lymphocytic inflammation as a consistent feature in peri‐articular, nongenetic human HO.^76^ In HO associated with cardiac valves, a polyclonal chronic inflammatory infiltrate is a common finding including lymphocytes, mast cells, and plasma cells.^173^, ^174^ Likewise, lymphocyte accumulation has been reported in the early stages of FOP lesions.^29^ Several lines of clinical or experimental evidence suggest that modulating lymphocytic inflammation may reduce HO genesis. For example, immunocompromised Rag1 mice (which lack B and T lymphocytes) demonstrate reduced HO formation after trauma.^175^ Likewise, corticosteroids inhibit experimental models of HO in mice.^135^ As previously mentioned, immunosuppressive corticosteroids are used clinically in FOP during flare‐ups to reduce HO formation.^24^, ^176^ There is also evidence that preoperative radiation in patients undergoing hip arthroplasty may change the inflammatory milieu of the area, thereby decreasing HO formation.^177^ Here, a study of hematoma fluid from patients undergoing total hip arthroplasty found that patients who had received preoperative radiation as HO prophylaxis had decreased numbers of T regulatory cells, increased frequency of cytotoxic T cells, as well as alterations in B‐cell maturation.^177^

#### Inflammatory cytokines

A number of studies have demonstrated altered levels of inflammatory cytokines associated with HO formation, both at a wound site or systemically. Most data suggest a positive correlation between increased inflammatory cytokines and HO formation. These data associate the degree of inflammatory response with the likelihood of HO genesis and may be used in the future in a predictive/diagnostic fashion. Correlation of levels of inflammatory cytokines to bone formation is best documented in nongenetic HO. For example, in a mouse model combining cutaneous burn with Achilles tenotomy, increased serum levels of TNFα, IL‐1β, IL‐6, and MCP‐1 were associated with HO formation.^178^ Local wound site expression of these markers as assessed by qPCR was generally found to temporally overlap with rise and fall of detected levels within the serum.^(178)^ Within the same model, increased levels of MCP‐1 were also found in murine saliva, although other assayed markers were too low for detection.^178^ Increased inflammatory markers have also been observed among human traumatic HO, both at the local and systemic level. For example, patients with penetrating, high‐energy extremity battle wounds demonstrated increased levels of several cytokines and chemokines that were individually associated with HO genesis.^179^ Specifically, serum IL‐6, IL‐10, and MCP‐1 and wound effluent IP‐10 and MIP‐1a were positively associated with HO formation.^179^ A similar study found that among combat‐associated high‐energy trauma, incidence of HO formation was associated with increased serum and wound effluent IL‐3.^180^ Conversely, serum IL‐12p70 and wound effluent IL‐13 in these patients were associated with a reduced likelihood of HO.^180^ It should also be noted that these clinical studies used a panel of 24 inflammatory cytokines and chemokines, and most did not correlate with HO incidence.

The correlation between inflammatory biomarkers and HO formation in FOP is less clear. In a case‐control study, the serum of 15 FOP patients was compared with 25 relatives to determine if increased tone of pro‐inflammatory cytokines could be detected.^181^ No statistically significant differences were found across 27 cytokines. However, two patients were reporting flare‐ups at the time of the sample collection and did trend toward higher G‐CSF and TNF‐alpha levels within their serum.^181^ In a genetic mouse model of HO (Nfatc1‐Cre/caAcvr1^fl/wt^, NFAT), transgenic mice demonstrated a mixed picture of systemic inflammatory markers.^178^ For example, some markers such as MCP‐1 showed increased levels of expression in both serum and saliva among transgenic animals compared with littermate controls. Other markers, such as IL‐1B or TNF‐alpha, showed an increase in saliva but not serum (or vice versa) among affected animals.^178^ The potential use of salivary biomarkers is an important question in FOP, as the minor trauma of a blood draw can trigger flare‐ups and HO.

Several major limitations exist in the evaluation of inflammatory cytokines. In the case, of traumatic HO, these patients represent complex clinical scenarios, often with multi‐system trauma who receive complex resuscitative, medical, and surgical interventions. As suggested in longitudinal assessments of the same patient, inflammatory cytokine content may vary widely with time. Moreover, from both mouse and human studies, it is clear that inflammatory cytokine content differs based on fluid analyzed (wound effluent, serum, or saliva), and even from the same patient at the same time these metrics do not always coincide. With these limitations considered, inflammatory cytokine studies in mouse and human suggest that increased local and systemic inflammation is associated with HO.

#### Trauma and inflammation

Traumatic injuries as well as burn and blast injuries are well‐characterized causes of increased systemic inflammation and predisposing factors to HO formation. These associations have been confirmed experimentally. One study to demonstrate this effect showed greater heterotopic bone formation when adipose‐derived stem/stromal cells were implanted in mice subjected to 30% body surface area burns.^78^ Conversely, traumatic HO has been reduced in experimental models by the application of apyrase at the injury site, which causes ATP hydrolysis and thereby reducing inflammation.^182^ Another study demonstrated that traumatic HO formation can be mitigated by the administration of rapamycin, which inhibits mTOR signaling and thereby alters the production of a number of inflammatory signals among other changes.^183^ Interestingly, targeting mTOR was also demonstrated to be effective in preventing HO formation in an FOP model.^184^

#### Neuroinflammation

As previously mentioned, CNS injury is a risk factor for HO genesis. The pathoetiologic links between the central and peripheral nervous systems and HO formation are not yet clear and likely are multifaceted. In animal models of FOP, blocking the sensory nerve pathway appears to decrease HO formation.^176^ As such, studies have examined the role of neuro‐inflammatory factors in HO formation, which may be dysregulated in trauma. Substance P has been observed to be increased in the lesions of both patients with FOP and with acquired HO.^185^ In a murine model of Achilles tendon HO formation, substance P delivery alone promoted HO formation and increased expression of BMP2; the addition of calcitonin gene‐related peptide (CGRP) in conjunction with substance P mitigated this effect. CGRP alone had little effect.^186^ Mast cells are recruited by sensory neurons by signaling factors such as substance P and have been strongly implicated in HO formation. When mast cell degranulation is prevented, less HO formation occurs.^167^ In a study of neurogenic HO, investigators have found substance P was elevated in the serum of neurogenic HO patients and that serum from neurogenic HO mice induced mesenchymal progenitor cells to undergo osteogenic differentiation in vitro.^100^ However, substance P is transient, and studies have not shown continued expression beyond the inflammatory phase of HO.

### Signaling pathways in HO

The exact signaling pathways responsible for HO formation have not been elucidated. Discussed below are several interacting pathways that have been identified as significant in this process.

#### BMP signaling

In 1965, Marshall R Urist discovered a substance in the extracellular bone matrix that had the ability to induce heterotopic bone when implanted in soft tissue.^187^ Since this time, BMPs have been implicated to have central roles in bone formation, bone repair, and HO. In fact, experimental models of HO often rely on application of recombinant BMP protein^140^, ^142^ or aberrant BMP2 or BMP4 overexpression.^142^, ^146^, ^188^ Models of trauma‐induced HO have observed increased BMP signaling, and BMP antagonism has reduced HO expanse in experimental models.^135^, ^189^ Although there is no single unifying signaling pathway responsible for both FOP and traumatic HO, the BMP pathway is important in both processes.^94^

As discussed, FOP is caused by mutations in the ACVR1 gene, which is thought to cause constitutive activation of the BMP type 1 receptor ALK2.^135^ Pathological activation of ACVR1 leads to overactivation of the BMP cascade. These studies have begun to change the thought of FOP as a process dependent on BMP ligands to one dependent on Activin A ligands. Supporting this observation, global expression of the R206H ALK2 receptor in animal models results in elevated levels of Smad 1/5/8 and in utero lethality.^190^ Evidence suggests that Activin A also plays a role in regulating innate immune cells and promoting the development of mast cells that are involved in the development of FOP lesions.^176^ Activin A neutralizing antibodies have shown remarkable inhibitory effects in FOP animal models.^191^ Ongoing clinical studies using an Activin A antibody offer hope to those living with FOP, although they are not likely to demonstrate efficacy in other forms of HO other than FOP.

#### Mammalian target of rapamycin (mTOR)

The mTOR signaling pathway regulates numerous cellular processes, including chondrogenic differentiation^192^ (see 193 for a review). Using cells from patients with FOP, aberrant Activin A signaling via the mutated ACVR1 receptor has been shown to increase mTOR signaling.^184^ Moreover, rapamycin has been shown to suppress HO formation in experimental FOP models.^151^, ^184^ In addition, administration of rapamycin inhibits experimental models of trauma‐induced HO formation.^151^, ^183^ The effect of the mTOR pathway in promoting HO formation appears to be augmented by leptin; in both in vitro studies on tendon‐derived stem cells and a rat model of traumatic HO formation via Achilles tenotomy, leptin promoted osteogenesis, and this effect was mitigated in the presence of rapamycin.^194^

#### Hypoxia‐inducible factors (HIFs)

HIFs activate genes encoding proteins that mediate adaptive response to reduced oxygen tension (see Laplante and Sabatini^195^ for a review). The HIF complex consists of 1 of 3 α subunits bound to HIFß. The HIFα pathway couples bone and vascular growth during development.^196^ Relative tissue hypoxia causes HIF1α activation, which increases production of pro‐angiogenic cytokines such as VEGF.^155^, ^197^, ^198^ Both experimental models of traumatic HO and FOP‐like HO demonstrate hypoxia and increased HIF1α signaling.^151^ FOP samples from human patients likewise show increased HIF1α immunostaining.^199^ Moreover, pharmacologic inhibition of HIF1α attenuates HO formation across experimental models of HO.^151^, ^199^ These results have been confirmed via alternative techniques, including gene silencing^200^ and genetic ablation.^199^

#### Retinoic acid receptor (RAR) signaling

Retinoids are potent morphogens, impacting both osteogenesis and chondrogenesis, to impact skeletal development.^201^ There are two classes of nuclear receptors, the retinoic acid receptors (RAR α, ß, and γ) and the retinoid x receptors (RXR α, ß, and γ). These receptors bind as RAR/RXR heterodimers or RXR homodimers to the DNA motifs called RA‐response elements (RAREs) to activate transcription of RA target genes.^202^ Unliganded receptors have also been recognized as having equally important function of actively repressing target gene repression through the recruitment of nuclear corepressors and associated histone deacetylases (HDACs).^(202,203)^ With regard to limb skeletal development, RARα is expressed throughout the limb mesenchyme early in limb development. As cells begin to differentiate into chondrocytes, RARα is downregulated, remaining highly expressed in the perichondrium, whereas RARγ expression becomes localized in the cartilaginous elements.^203^, ^204^

It was previously demonstrated that the continued expression of RARα in prechondrogenic cells prevents their differentiation, resulting in skeletal developmental malformations in transgenic mice,^205^ leading to the idea that RARα agonism could impede HO formation. A selective RARα agonist was first tested in a subcutaneous rBMP2‐induced HO model in mice.^206^ Reduced amounts of heterotopic cartilage and bone were found, yet complete abrogation of HO was not found.^125^, ^206^ Follow‐up studies examined the effect of both RARα and RARγ agonists.^125^ RARγ agonists were observed to abrogate rBMP2‐induced subcutaneous and intramuscular HO, as well as dramatically reduce ossification in the ALK2^Q207D^ mouse model.^125^ Both agonists for RARα and RARγ inhibited chondrogenesis and prevented HO, but RARγ agonists were more effective.^134^ The higher efficacy of RARγ agonists may be explained by a wider expression pattern of RARγ in both chondrogenic cells and chondrocytes compared with RARα and RARß.^125^, ^134^ Mechanistically, RARγ agonists have been observed to dampen BMP signaling, both by inhibition of Smad phosphorylation and by a significant proteasome‐mediated drop in overall Smad levels.^125^ Palovarotene, a specific RARγ agonist (which was previously tested in a clinical trial for emphysema) was shown to counteract multiple soft tissue and skeletal pathologies in Acvr1^R206H/+^ mutant mice. In their damaged muscle tissue, Palovarotene was associated with fewer mast cells and reductions in fibroproliferation, cartilage formation, and the amount of HO.^134^ Recent studies in animal models have suggested potential off‐target effects of RARγ agonist treatment, including skeletal abnormalities and delayed wound healing.^207^, ^208^ Nevertheless, ongoing clinical trials of the RARγ agonist Palovarotene have shown potential for the treatment of FOP.^209^ Palovarotene is currently being studied in a phase 3 clinical trial.^210^

#### GNAS

GNAS is a complex imprinted gene of the alpha‐subunit of the stimulatory heterotrimeric G protein (Gα_S_). As a key regulator in skeletal development, Gα_S_ inhibits hedgehog (Hh) signaling and loss of GNAS results in aberrant Hh signaling activation.^211^ As previously discussed, inactivating mutations within the GNAS gene results in either Albright's hereditary osteodystrophy (AHO) or POH (progressive osseous heteroplasia). HO formation resembling POH has been replicated experimentally in mouse models using several Cre lines, including Prx1‐Cre, Dermo1‐Cre, and Ap2a‐Cre.^211^ In these animal models and in POH human samples, Hedgehog signaling activity is increased. Moreover, Hh signaling pathway inhibition, either by genetic or chemical approaches, reduced HO formation in this animal model.^211^ Somewhat analogous findings regarding the presence of Hh signaling activation have been observed in predominantly endochondral HO mouse models as well.^212^ For example, in a BMP4 overexpression model of mouse model of HO, Gli1‐expressing cells (using the Gl1‐CreERT reporter system) were found to contribute to all stages of endochondral HO.^212^

## Summary

In summary, heterotopic ossification is a diverse pathologic process, with different etiologies, tissue locations, mechanisms of ossification, and putative cell types of origin. Uniting this diversity of HO are key commonalities, including the phasic nature of the disease process that often arises in a background of inflammation with or without tissue trauma. Those susceptibility factors that predict which patients and what injury types will progress to HO formation are not well defined. The extent to which inflammatory cascades can be manipulated to prevent HO formation is only beginning to be understood. Moreover, current prophylactic or treatment strategies have significant shortcomings. These gaps in our knowledge require further study of this distinctive and understudied condition.

## Disclosures

All authors state that they have no conflicts of interest.
